# The Frequency of Intraventricular Hemorrhage and its Risk Factors in Premature Neonates in a Hospital’s NICU

**DOI:** 10.22037/ijcn.v15i3.21592

**Published:** 2021

**Authors:** Behnaz BASIRI, Mohammad Kazem SABZEHEI, Maryam SHOKOUHI SOLGI, Elham KHANLARZADEH, Mojdeh MOSHEIRI

**Affiliations:** 1 Department of neonatology, Faculty of Medicine, Hamadan University of Medical Sciences, Hamadan, Iran; 2 Department of Social Medicine, Hamadan University of Medical Sciences, Hamadan, Iran

**Keywords:** Intra-ventricular hemorrhage, premature neonate, low birthweight, Pneumothorax

## Abstract

**Objective:**

Intra-ventricular hemorrhage (IVH) is the leading cause of mortality and disability in premature neonates. The present study aimed to determine the frequency of IVH and its risk factors in the premature newborns admitted to the Neonatal Intensive Care Unit (NICU)at Fatemieh Hospital in Hamadan, Iran, in 2016.

**Methods & Materials:**

This retrospective cross-sectional study was conducted on178 neonates with a gestational age of ≤ 32 weeks admitted to Fatemieh Hospital affiliated to the Hamadan University of Medical Sciences, Hamadan, Iran, in 2016. The study population was selected using the census method. The newborns were subjected to cranial ultrasound on the seventh day of life, and they were assigned into two case and control groups (namely neonates with IVH and those without IVH, respectively). Intra- ventricular hemorrhage was classified into four grades regarding Papile classification. The patients’ demographic specifications, including 1- and 5-minute Apgar scores, type of delivery, birth weight, use of mechanical ventilation, prenatal corticosteroid, gestational age, and some complications (e.g., Pneumothorax), were collected using a checklist. The data were analyzed using SPSS software version 16.

**Results:**

According to the results, the prevalence of IVH in premature infants admitted to NICU was approximately 20%, and 61.2% of the neonates were male. The participants’ mean gestational age was 30.39 weeks. The comparison of delivery type between the case and control groups revealed no significant difference (*P*=0.197). Furthermore, there was a significant difference between the two groups in terms of their need for mechanical ventilation (*P*=0.03), Pneumothorax(*P*=0.001), and 5-minute Apgar scores (*P*=0.04). Moreover, the incidence of IVH had a significant relationship with the mean gestational age (*P*=0.001) and birth weight (*P*=0.04).

**Conclusion:**

According to the findings, the premature newborns admitted to the NICU revealed a relatively high prevalence of IVH. The condition is aggravated in preterm neonates by some factors such as low birth weight, 5-minute Apgar score, gestational age, and the need for mechanical ventilation.

## Introduction

Intraventricular hemorrhage (IVH) is one of the leading causes of mortality and disability in premature newborns, leading to motor disorder, paralysis, long-term cognitive impairment, mental retardation, and seizure[1].

Several studies conducted worldwide during the past five years have reported the incidence rate of IVH to be between 20%-40% in preterm infants. The incidence of IVH has decreased significantly over the last decade due to the global improvements in neonatal care, which seems to be because of improving practices such as the use of antenatal corticosteroids, practical resuscitation skills, appropriate handling of infants, better infrastructure, and the judicious use of ventilation [2-5]. However, an increase in preterm delivery resulting from the enhancement of assisted reproductive technology-mediated pregnancy has highly affected the incidence rate of IVH[3].

IVH is the extension of hemorrhage into the lateral, third, and fourth ventricles (6) and is less prevalent among full-term neonates because they have a more complex germinal matrix. IVH can emerge in the fetus, and the diagnosis of fetal IVH is based on antenatal ultrasonography and MRI. Postnatal survival is high; however, the neurodevelopmental delay is probable (7) 

The risk factors for GMH(Germinal Matrix Hemorrhage)-IVH are acute inflammation of the placenta, increased number of leukocytes in the first 72 h after birth, increased white blood cell count, and masculinity[4]. The other risk factors for IVH include prematurity, low birthweight (LBW), long term mechanical ventilation, low 5-minute Apgar score, hypoxia-induced damage, hypothermic ischemia, Pneumothorax, and thrombocytopenia, antenatal maternal hemorrhage, maternal infection/ inflammation, sepsis, hypotension, hypoxia, hypercapnia, seizures, patent ductus arteriosus (PDA), infection, and respiratory distress, genetic factors(2,6). 

IVH cannot be diagnosed clinically as such neuroimaging is necessary. According to the Quality Standards Subcommittee of the American Academy of Neurology and the Practice Committee of the Child Neurology Society, routine screening cranial ultrasonography (CS) should be performed in all preterm infants with <30 gestation weeks once during their 7-14 days of age. Other diagnostic tools such as transcranial Doppler ultrasonography, near infra-red spectroscopy (NIRS), and advanced MRI techniques are recently being used to provide insight into the brain vascular anatomy and hemodynamic information. They also contribute to the prognosis of long-term neurodevelopmental outcomes(2).

About 15-20% of premature newborns are exposed to IVH, and this is associated with serious complications and mortality in the absence of timely diagnosis and intervention (8). Since preterm newborns’ brain is sensitive to blood pressure fluctuations, routine care measures seems to be essential among this population (9). 

Recently, a series of measures (namely cesarean delivery, delayed cord clamping, minimal handling of infants, avoiding head down position, midline head positioning for 72h, keeping head of the bed up at 15 - 20 degrees, slow infusion of fluids, and antenatal corticosteroids for IVH prevention) have been adopted in different NICUs(2).In a study examining the effects of delayed cord clamping on IVH in preterm infants, the incidence rates of IVH and periventricular leukomalacia (PVL) were 11.43% and 5.7% in the early clamping group, respectively. In the delayed clamping group, the rate was 0%. Accordingly, they concluded that delayed cord clamping might be used as a reliable technique in reducing the IVH rate (10).

The findings of a systematic review addressing the effectiveness of head midline position in preventing the occurrence or extension of GMH-IVH in preterm infants are consistent with those dealing with the beneficial or detrimental effects of a supine head midline position and do not provide a single response to the review question (11).

Given the high complication of IVH and its high mortality rate in preterm newborns, and the lack of sufficient research in this regard, the present study aimed to determine the frequency of IVH and its risk factors in the premature neonates admitted to NICU at Fatemieh Hospital in Hamedan, Iran.

## Materials &Methods

This retrospective cross-sectional study was conducted on 178 premature neonates (with a gestational age of ≤32 weeks admitted to NICU at Fatemieh Hospital affiliated to the Hamadan University of Medical Sciences, Hamedan, Iran, in 2016. The study population was selected using the census method, and patients who were discharged or died before the seventh day of birth were excluded from the study. 

***Study Design***

The demographic data were extracted from the neonates’ medical records, which encompassed gestational age, birth weight, type of delivery, use of mechanical ventilation, 1- and 5-minute Apgar scores, head circumference, Pneumothorax, thrombocytopenia, resuscitation, prescription of corticosteroid for mother before delivery, and neonatal and maternal profiles. The newborns were assigned into two case and control groups (namely newborns with IVH and those without IVH, respectively).

Cranial ultrasound was performed on the seventh day of birth with the emergence of intra-ventricular hemorrhage symptoms such as reduced blood pressure, apnea, jaundice, cyanosis, weak suck reflex, abnormal ocular symptoms, high-pitch sounds, semi-screaming crying, seizure or weak muscle tone, metabolic acidosis, shock, and decreased hematocrit. A radiologist performed all ultrasounds using the GE Volson E6 device.

The IVH diagnosis was accomplished based on ultrasound studies using Papile classification, according to which the severity of this condition was classified into four grades: Grade 1(slight bleeding in the sub-ependymal germinal matrix), Grade 2(extensive bleeding in <50% of the ventricular and intracerebral hemorrhage without ventricular dilatation), Grade 3(extensive bleeding in >50% of the ventricular and intraventricular hemorrhage with ventricular dilatation), and Grade 4(periventricular venous infarction involving the obstruction of blood flow through the periventricular terminal vein).

***Statistical analysis***

In this study, the independent t-test was used to compare the quantitative risk factors such as a 5-minuteApgar score, birth weight, and gestational age at birth between the two groups. Furthermore, the Chi-square test was also performed to compare the two groups in terms of qualitative variables such as type of delivery, thrombocytopenia, use of mechanical ventilation, gender, resuscitation, maternal consumption of corticosteroid before delivery, Pneumothorax, and decreased and increased blood pressure. The data were analyzed using SPSS software version 16. P< 0.05 was set as the significance level. 

***Ethical considerations***

*This study was extracted from a thesis submitted in the partial fulfillment of a requirement for the degree of MD.* *The present research was approved by the Ethics Committee of the Hamadan University of Medical Sciences and recorded at the clinical trial center. *The confidentiality and anonymity of the participants’ data were observed.

## Results

According to the results of this study, 61.2% of the neonates were male, and their mean gestational age was 30.39±1.71 weeks (range: 24-33 weeks). Furthermore, the participants’ mean birth weight was 1542.33±354.55 g (range: 659-2600 g). About 21.4% and 87.6% of the infants were born via vaginal delivery and cesarean section, respectively. Moreover, 52.8% of the newborns were exposed to neonatal resuscitation at birth, while 47.2% of the participants needed no resuscitation. Almost 73% of the neonates received mechanical ventilation, and the rest had spontaneous breathing. Pneumothorax was observed in only 2.3% of the newborns, and thrombocytopenia was noticed in 5.6% of the neonates.

The mean 1- and 5-minute Apgar scores were 5.65±1.83 (range: 1-9) and 7.74±1.45 (range: 2-10). The mean head circumference was 29.33±2 cm (range:26-34). In general, about 21.4% (n=38) of the newborns had IVH. Among the newborns with IVH, 55.3% (n=21), 21.1% (n=8), 13.2% (n=5), and 10.5 % (n=4) had types I, II, III, and IVH, respectively. Table 1 presents the frequency of gender, type of delivery, neonatal resuscitation, use of mechanical ventilation, Pneumothorax, and thrombocytopenia regarding IVH. 

According to Table 1, the two groups are comparable in terms of gender (*P*=0.305), and there is no significant difference between the case and control groups regarding the type of delivery (*P*=0.197). Comparing the two groups in terms of resuscitation revealed no significant difference (*P*=0.98). Likewise, no significant difference was observed between the two study groups in terms of thrombocytopenia (*P*=0.138). The case and control groups differed significantly regarding the need for mechanical ventilation (*P*=0.03). Moreover, the comparison of Pneumothorax between the newborns with and without IVH indicated a significant difference (*P*=0.001). 

Table 2 shows the mean gestational age, weight, 1- and 5-minute Apgar scores of IVH.As it can be observed, IVH revealed a significant relationship between the mean gestational age and neonatal weight(*P*=0.001&*P*=0.04, respectively). The comparison of head circumference and 1-minute Apgar scores between the case and control groups indicated no significant difference (*P*=0.201&*P*=0.293, respectively).However, there was a significant difference between the two groups in terms of the 5-minuteApgar scores (*P*=0.04).

## Discussion

Our findings showed that the premature newborns with IVH had lower mean gestational age, weight, and 5-minute Apgar scores than other newborns. Furthermore, all newborns with IVH were inflicted with Pneumothorax, 85% of whom needed mechanical ventilation. In the present study, about one-fifth of all premature neonates had IVH, and this finding is consistent with the rate reported in a similar study in Iran (12). In a study by Philip et al., IVH was diagnosed in nearly 20% of the preterm newborns with the gestational age of 34 weeks(13). 

The prevalence of IVH was estimated to be 36% in newborns with the gestational age of22-28 weeks(14). In addition, in a study by Dyet et al., brain hemorrhage was observed in more than 40% of the infants born during the 23rd-30th weeks of gestation, and ventricular dilation was reported in half of these neonates after IVH(15). In another study, the prevalence rate of GMH/IVH was45%for premature neonates [3].Furthermore, the prevalence rate of IVH was estimated to be>60% in Sajjadian’s et al. research in Iran(16). There are different reports on the prevalence of IVH among premature neonates, addressing some factors such as the time and place of study.

In our study, a majority of the newborns with IVH had grade I, and less than a quarter of the newborns suffered from grade II and III hemorrhage. The prevalence rates of grades II, III, and IV IVH were higher in Sajjadianas' study compared to those obtained in the present study (16). Furthermore, there was a direct relationship between the IVH grade and patient prognosis. In this regard, Brouwer et al. demonstrated that the prognosis of premature newborns with grade III (VH) was better than those with grade IV IVH (17). 

Bolisetty et al. showed that the developmental delay, cerebral palsy, deafness, and blindness were more in newborns with higher degrees of IVH (18). Moreover, the probability of survival in the neonates suffering from IVH with a higher hemorrhage grade was also better than that of the newborns with lower hemorrhage grades (19). In the present study, gestational age was the main factor affecting the incidence of IVH, and this finding is in line with those of other similar studies (14, 20-22). 

The IVH degree has a strong relationship with the incidence of complications in brain evolution; therefore, it has a reverse relationship with gestational age and birth weight. Accordingly, the younger newborns and those with lower weight are at higher risk of IVH than the others (4).In accordance with our findings, Mulindwa et al. reported that the mean weight and gestational age of the neonates with IVH were lower than those without this problem. Their findings were in line with those of our study regarding the frequency of IVH and its degrees among newborns (23).

In our study, the mean neonatal weight was about 125 g higher in the case group than in the control group, indicating an association between LBW and IVH. This finding is in agreement with the findings of the other similar studies (24). Szpecht et al. concluded that the birth weight of the premature newborns was lower than that of other infants (22). Furthermore, Moghaddam et al. demonstrated that most premature neonates had LBW, and that IVH was observed in less than 10% of LBW neonates [26]. Newborns with a weight <1000 g have a high risk of IVH (about 14% and 24% for 750-1000 and ≥750 g, respectively). 

However, grades III and IV hemorrhage were reported in 7.1% of the premature neonateswith the weight of 1000-1500 g (6). In this regard, IVH is the main reason for mortality among premature newborns worldwide, and about 30% of premature newborns with weight below 1500 g suffer from IVH. Our findings revealed that 85% of the premature newborns who needed mechanical ventilation had IVH. Premature newborns are more likely to need mechanical ventilation for survival since they are at a higher risk of IVH than their full-term counterparts. 

According to a study performed by Graziani et al., mechanical ventilation on the first day of life was associated with gestational age, birth weight, Apgar scores, and grades III/IV intracranial hemorrhage (26). Similarly, it was reported that the premature newborns had a higher5-min Apgar score, a need for mechanical ventilation, and Pneumothorax prevalence(12). 

The effect of delivery type on the occurrence of IVH has been disregarded in the literature. In the present study, comparing the type of delivery between the newborns with and without IVH showed no significant difference in this regard. However, there are inconsistent findings in this regard (1, 12, 27, 28); therefore, further studies are recommended to investigate this issue. In Humberg’s et al. study, no significant relationship was observed between the type of delivery and the incidence of IVH(29).

In general, some main risk factors for IVH are LBW (1), gestational age(12,20), 5-minute Apgar score(12, 22,26), in vitro fertilization (1,27), mechanical ventilation duration(12), and type of delivery (12,27).

In some rare cases, IVH is diagnosed immediately after birth. In this regard, only 50% of newborns are diagnosed on the first-day after delivery. Accordingly, 75% and 90% of the neonates are diagnosed in the first three days and the first week of their birth, respectively(30), while less than 5% of the newborns are diagnosed after four or five days following their birth(31). The incidence of IVH after one month of birth is a rare event; however, some patients with IVH have no clinical symptoms(6). 

According to the American Academy of Neurology recommendation, younger gestational age is associated with IVH; hence, screening preterm neonates aged below 30 gestational weeks is of paramount importance ( 31). According to Nocik et al., most of the IVH diagnoses are based on the IVH risk factors, and only a small percentage of neonates are diagnosed by ultrasound techniques (32).

This study aimed to examine the frequency and risk factors for IVH in NICU and to detect what reduces the incidence of IVH. According to this study, the frequency of IVH in our NICU is not higher than that in other centers in Iran, and the detected risk factors are similar to those in other studies. One of the limitations of the present study to be addressed in future studies, no follow-up period was considered for our IVH patients.

**Table 1:** The frequency of risk factors based on intraventricular hemorrhage

Variables		With intraventricular hemorrhage (n=38)		Without intraventricular hemorrhage (n=140)		P-value (Chi-square test)
		No	Percent	No	Percent	
Gender	Male	26	68.4	83	59.3	0.305
	Female	12	31.6	57	40.7	
Type of delivery	Natural	11	28.9	27	19.3	0.197
	Cesarean section	27	71.1	113	80.7	
Resuscitation	Yes	20	52.6	74	52.9	0.98
	No	18	74.4	166	47.1	
Mechanical ventilation	Yes	33	86.8	97	69.3	0.03
	No	5	13.2	43	30.7	
Pneumothorax	Yes	4	10.5	0	0	0.001
	No	34	89.4	140	100	
Thrombocytopenia	Yes	4	10.5	6	4.3	0.138
	No	34	89.4	134	95.7	

**Table 2:** The participants’ demographic information by intraventricular hemorrhage

Variables	With intraventricular hemorrhage		Without intraventricular hemorrhage		P-value (t-test)
	Mean	SD	Mean	SD	
Gestational age	29.5	2.35	30.64	1.41	0.001
Weight	1423.42	422.14	1574.6	328.2	0.04
Head circumference	28.68	3.76	29.51	2.11	0.201
1-minute Apgar scores	5.36	1.86	5.72	1.83	0.293
5-minute Apgar scores	7.23	1.8	7.87	1.31	0.04

## In Conclusion

IVH is a severe multifactorial complication in premature newborns, which is caused by neonatal prematurity and underweight. According to the present study's findings, IVH had a relatively high prevalence in neonates admitted to the NICU in Hamadan. Furthermore, the risk of this condition in preterm neonates is enhanced by some factors such as low LBW, 5-minute Apgar score, gestational age, and the need for mechanical ventilation. According to the low Apgar score, it is necessary to focus on treating neonatal resuscitation programs as a prominent IVH risk factor.
